# Psychometric properties of the job anxiety scale

**DOI:** 10.3389/fpsyg.2023.1020596

**Published:** 2023-04-25

**Authors:** Ileana Schmalbach, Bjarne Schmalbach, Andreas Kalkbrenner, Markus Bassler, Andreas Hinz, Katja Petrowski

**Affiliations:** ^1^University Medical Center of the Johannes Gutenberg University Mainz, Mainz, Germany; ^2^Dresden University of Technology, Carl Gustav Carus Medical Faculty, Department of General Medicine/MK3, Dresden, Germany; ^3^Hochschule Nordhausen, University of Applied Sciences, Nordhausen, Germany; ^4^Department of Medical Psychology and Medical Sociology, University of Leipzig, Leipzig, Germany

**Keywords:** job anxeity, health outcome, psychometric properties, psychology, job worries

## Abstract

**Background:**

Occupational stress and specifically job anxiety are crucial factors in determining health outcomes, job satisfaction as well as performance. In order to assess this phenomenon, the Job Anxiety Scale (JAS) is one of the instruments available. It consists of 70 items that are clustered in 14 subscales and five dimensions. This manuscript is a revised version of a retracted article that analyzed the properties of a short version of the JAS. Rather than shortening the scale, the authors of the JAS recommend to further assess the scale in its current state without modification of the factor structure. Hence, the aim of this paper is to assess the psychometric properties of the original JAS.

**Methods:**

The sample consists of 991 – mostly psychosomatic – patients from two different clinics. We applied methods of factor analysis and bivariate correlations to explore and test factor structure and the nomological net of related constructs.

**Results:**

The Job Anxiety Scale evinced satisfactory psychometric properties. We found very high internal consistency, and invariance across participant age. It displayed good discriminant validity and we found the expected pattern of convergent correlations. However, the model fit is not convincing.

**Conclusion:**

With the Job Anxiety Scale, researchers can assess job related worries in a reliable manner. The questionnaire is particularly useful in large-scale surveys, in therapy or work-related contexts. However, the scale could be modified in order to aim for a better fit and assess job related anxiety in a more efficient way.

## Background

Occupational stressors are crucial predictors in explaining a wide range of positive and negative job-related outcomes. For instance, higher levels of job-related stress and anxiety are associated with lower levels of job satisfaction, commitment to the job, and burn out (Borg et al., 1991; Newbury-Birch and Kamali, 2001; Wu et al., 2021; Yang et al., 2021).

Further, job-related stress and anxiety is negatively linked to social support and well-being (Abdel-Halim, 1982; Warr, 1990; Dormann and Zapf, 2002; Greenglass, 2002; Huhtala et al., 2021) and positively related to adverse mental health outcomes (Hobson and Beach, 2000; Muschalla et al., 2013).

Recent and past researches (Motowidlo et al., 1986; Karatepe, 2012) have even suggested an impact on performance based on the frequency of stressful experiences. It is estimated that almost 5% of German employees are at risk of being absent from work due to job anxiety (Muschalla et al., 2013). This fact is associated with high costs for companies, health insurance and public pension funds. Thus, research into the phenomenon and a reliable and valid assessment of the construct are of great interest to researchers and practitioners alike. Even more in the face of the ongoing covid-19 pandemic, which can trigger and worsen job anxiety (Probst et al., 2020).

Individuals who experience job-related anxiety experience typical anxiety symptoms such as trembling, blushing or palpitations when they are at work or when they think about their work (Muschalla et al., 2010). Even though these symptoms have a major influence on work performance, there is no ICD code for workplace phobia. Haines and colleagues (Haines et al., 2002) used the criteria of phobia to characterize workplace related anxieties: Intense anxiety when approaching the workplace, incapacity to enter the workplace because of anxiety, respectively, panic symptoms and a reduction of anxiety when leaving the workplace. Bryson and colleagues (Bryson et al., 2013) implemented of parts of Warr’s anxiety-contentment axis model (Warr, 2007) for measuring job anxiety. Even though it is a clinically and economically important construct, there is only one questionnaire available for measuring job anxiety in a comprehensive manner – the Job Anxiety Scale (Linden et al., 2008).

Muschalla (2005) ran a pilot study, using an initial version of the JAS with 106 items. This version contained criteria of anxiety related to ICD-10 (World Health Organization, 1992), DSM-IV (American Psychiatric Association, 1994) and job-related anxieties often reported by patients. Based on this first trial, Linden and colleagues (Linden et al., 2008) modified the scale into its current version with 70 items. The items are clustered by theoretical assumptions in five dimensions. The five dimensions assess issues related to *stimulus-related anxiety* and *avoidance behavior*, *social anxieties* (e.g., bullying), *insufficiency* (e.g., Low self-esteem), *health-and body-related anxieties*, as well as *job-related worrying*. In addition, two items measure a *global workplace-anxiety*. For the assessment of job-related anxiety each subscale and dimension as well as a global mean value can be analyzed based on the 70 JAS items. Its psychometric properties, such as internal consistency (Linden et al., 2008; *α* = 0.98) and retest-reliability (Linden et al., 2008; Muschalla et al., 2013) (82–0.85) are good. The scale correlated (*r* = 0.67–0.69) with the Stait-Trait Anxiety Inventory (Spielberger, 1983) (STAI-T) illustrating its convergent validity (Linden et al., 2008; Muschalla et al., 2013). Nonetheless, the factorial structure of the scale has not been yet assessed until now.

The *Workplace Phobia Scale* (Muschalla and Linden, 2008) (WPS) screens for job anxiety based on 13 items. These items were selected from the JAS rather than being empirically driven. The authors used all items from the subscale’s *stimulus-related anxiety* and *avoidance behavior* and two items of the *global workplace-anxiety* subscale. Since stimulus-related anxiety and avoidance behavior are the central aspects of phobia, the WPS captures phobia and criteria of clinic disorders. Even so, the WPS does not allow a wide non-phobia, clinic-specific, comprehensive assessment of job anxieties in the manner the JAS does. Specifically, cognitions of bullying and insufficiency as well as health-related thought patterns are not captured. Thus, the aim of the present study is to target this gap. Furthermore, as job anxiety is not an ICD diagnosis or clinical diagnosis, there is a need for a statistically sound scale that measures work-related worries in a comprehensive construct. Especially for surveys in non-clinical and work-related contexts a validated scale to measure job anxieties could be of great relevance. A primary relevant application relies in the field of occupational psychological and practice and for instance, the evaluation of risk assessment of unemployment in the context of medical rehabilitation. Concerning the latter, general performance restrictions due to illness, fears and low self-esteem are often directly related to the workplace.

This manuscript is a revised version of a retracted article that analyzed the properties of a short version of the JAS (Schmalbach et al., 2020)^.^ Rather than shortening the scale, Muschalla (2005) recommend to further assess the scale in its current state without modification of the factor structure. Hence, the aim of the study at hand is to analyze item and scale properties, conducted factor analysis and tests of measurement invariance. In addition, to determine convergent and discriminant validity, we examining its associations with a measure of psychosocial health, the HEALTH-49 (Rabung et al., 2007, 2009). To this end we expect significant moderate to high correlations between the subscales of the HEALTH-49 related to psychological and somatoform symptoms, difficulties in interactions and social distress and the JAS. On the other hand, we hypothesize sig. and negative associations between scales related to psychological well-being, self-efficacy, activity participation and social support. Based on the theoretical background of the scale we propose a 5-factor model that illustrates the theoretical assumptions of the scale and invariance for sex and age.

## Method

### Study sample

We recruited a German study sample in the Clinic of Psychotherapy and Psychosomatic Medicine, University Hospital Dresden (*n* = 284) and in the Rehabilitation Center Oberharz (*n* = 758). We focused on patients and individuals in rehabilitation because, first, the JAS was developed in a similar setting (Linden et al., 2008), and second, such a sample (vs. a general population sample) will yield a broader distribution of the characteristic in question.

#### Description of the study sample of the participants in the University Hospital Dresden

We included *n* = 169 females (59.5%) with a mean age of *M* = 36.64 (*SD* = 13.19) years and 115 males (40.5%) with a mean age of *M* = 37.39 (*SD* = 12.20) years. The overall mean age of the sample was *M* = 36.94 (*SD* = 12.88; range, 17–83) years. 26.7% of the sample lived alone in their household, 71.5% of the sample lived together with one or more people. The diagnoses for this group are displayed in Table 1.

**Table 1:** Diagnoses of both subsamples.

	Frequency	%
Diagnoses of the Patients from the Technische Universität Dresden, University Hospital Carl Gustav Carus (*n* = 277).		
F40-48 – Neurotic, stress-related and somatoform disorders	151	54.5
F30-39 – Mood (affective) disorders	89	32.1
F50-59 – Behavioural syndromes associated with physiological disturbances and physical factors	4	1.4
F10-19 – Mental and behavioural disorders due to psychoactive substance use	3	1.1
Other diagnoses	30	10.8
Diagnoses of the Inpatients from the Rehabilitation Center Oberharz (n = 758)		
F40-48 – Neurotic, stress-related and somatoform disorders	387	51.1
F30-39 – Mood (affective) disorders	304	40.1
M50-54 – Other dorsopathies	35	4.6
F60-69 – Disorders of adult personality and behaviour	8	1.1
Other diagnoses	24	3.2

#### Description of the study sample of the participants in Oberharz

The second group (*N* = 758) consists of patients from the “Rehabilitation Center Oberharz” (Rehazentrum Oberharz). Four hundred eleven females (54.2%) with a mean age of *M* = 46.90 (*SD* = 8.66) years and *n* = 347 males (45.8%) with a mean age of *M* = 47.14 (*SD* = 9.99) years were assessed in this sample. The overall mean age of the sample was *M* = 47.01 (*SD* = 9.29; range, 18–74) years. The diagnoses for this group are also displayed in Table 1.

All participants signed an informed consent form and received a data protection declaration in agreement with the Helsinki Declaration. The study was approved the ethics committee of the Medical Faculty of the Technische Universität, Dresden (EK 79032011). Verbal and written informed consent were obtained from all participants.

### Instruments

The JAS questionnaire (Linden et al., 2008) consists of five dimensions, 14 subscales and 70 items. Each item was scored on a 5-point Likert scale, ranging from of 0 (no agreement) to 4 (full agreement) – with no reverse-scored items. The scale shows an excellent reliability (Linden et al., 2008) (Cronbach’s *α* = 0.96).

We used the *Hamburg Modules for the Assessment of Psychosocial* Health (Rabung et al., 2007, 2009) (HEALTH-49) to measure general psychosocial well-being and health in the respondents. The scale comprises 49 items to assess nine subscales (and a psychological symptoms aggregate), which include mental health symptoms, self-efficacy, well-being, as well as social support and participation. As per Rabung et al. (2009) internal consistency is acceptable to very good for all subscales with values between *α* = 0.73 and 0.91.

### Statistical analyses

All analyses were performed in R, using the packages *lavaan* (Rosseel, 2012). Missing values were replaced by linear interpolation up to a limit of 5% missing values. Data sets containing more than 5% missing values were deleted. To test the above-mentioned hypothesis concerning the factorial purpose, we conducted a confirmatory factor analysis using *lavaan* employing a robust maximum likelihood estimation (Satorra and Bentler, 2001) and robust formulas for the estimation of fit indices (Brosseau-Liard and Savalei, 2014). To evaluate model fit, we applied the commonly recommended indicators and cutoffs (Hu and Bentler, 1999; Schermelleh-Engel et al., 2003): *χ*^2^-test (non-significant), χ^2^/*df* (<2), Comparative Fit Index (*CFI* > 0.95), the Tucker-Lewis Index (*TLI > 0.95*), the Root Mean Square Error of Approximation (*RMSEA* < 0.08), and the Standardized Root Mean Square Residual (*SRMR* < 0.08). We report reliability as McDonald’s ω, which is the preferred measure of internal consistency (Dunn et al., 2014).

For the investigation of measurement invariance, we used the common step-wise model comparison approach (Meredith, 1993). In this procedure, one compares increasingly restrictive models to establish increasingly strict levels of invariance. Specifically, the first step is the comparison of the configural (unconstrained) model with the metric (equal factor loadings across compared groups) model. Second, one compares the metric to the scalar (equal item intercepts across compared groups) model. Finally, one compares the scalar to the strict (equal residual terms across compared groups) model. To evince measurement invariance, χ^2^ should not be significant and the difference in *CFI* and gamma hat (*GH*) should not exceed 0.01 (Milfont and Fischer, 2010). In order to determine convergent and discriminant validity we conducted Pearson’ product–moment correlations between the scales of the JAS and HEALTH-49.

## Results

The sample distribution showed a slightly pronounced left-skewedness, but the descriptive statistics were satisfactory for most of the JAS items (see Table 1); with some exceptions (item 31,53, 59, 69, 70) factor loadings exceeded 0.50 (0.513 ≤ λ ≤ 0.888), and reliability coefficients were between ω = 0.843 and 0.956 for the five subscales (see Table 2). The confirmatory analysis, revealed a 5-factor structure with unacceptable fit χ^2^(2335) = 12581.35, *p* < 0.001, *CFI* = 0.753, *TLI* = 0.744, *RMSEA* = 0.078, *SRMR* = 0.064. For exploratory purposes, we then tested a unidimensional model, showing a less acceptable fit, χ^2^(2345) = 14649.624, *p* < 0.001, *CFI* = 0.700, *TLI* = 0.691, *RMSEA* (90% *CI*) = 0.086, *SRMR* = 0.06, despite its very high internal consistency of *ω* = 0.976.

**Table 2:** Descriptive statistics of the JAS-70 items and scales.

Item-Nr.	Dimension	*M*	*SD*	γ1	γ2	λ
1	C	1.751	1.402	0.335	1.83	0.831
2	A	1.83	1.447	0.235	1.713	0.794
3	C	2.303	1.319	−0.147	1.814	0.693
4	D	1.587	1.429	0.45	1.84	0.758
5	A	1.399	1.467	0.625	1.953	0.796
6	A	1.762	1.476	0.269	1.667	0.687
7	C	1.911	1.488	0.15	1.605	0.674
8	D	1.828	1.406	0.229	1.735	0.772
9	A	1.382	1.445	0.627	1.975	0.743
10	A	0.798	1.323	1.504	3.816	0.775
11	D	1.405	1.393	0.655	2.133	0.774
12	D	1.852	1.427	0.195	1.702	0.852
13	D	1.505	1.392	0.556	2.014	0.749
14	E	1.491	1.399	0.537	1.993	0.609
15	B	1.307	1.277	0.755	2.497	0.569
16	D	1.104	1.172	0.975	3.071	0.679
17	D	1.071	1.216	1.04	3.104	0.505
18	D	1.654	1.405	0.381	1.852	0.856
19	A	1.642	1.479	0.425	1.767	0.752
20	E	1.713	1.342	0.382	1.961	0.75
21	C	1.572	1.408	0.492	1.934	0.89
22	A	0.804	1.248	1.484	3.944	0.739
23	A	0.94	1.368	1.219	3.043	0.808
24	B	1.588	1.46	0.534	1.908	0.451
25	B	1.188	1.407	0.917	2.459	0.786
26	B	1.251	1.423	0.821	2.271	0.784
27	B	0.82	1.095	1.479	4.524	0.63
28	A	1.235	1.488	0.85	2.213	0.694
29	C	1.548	1.463	0.526	1.88	0.851
30	A	1.242	1.448	0.832	2.255	0.855
31	B	0.713	1.114	1.674	4.917	0.497
32	C	1.018	1.331	1.129	2.993	0.781
33	A	1.143	1.389	0.945	2.528	0.761
34	C	1.338	1.431	0.716	2.122	0.711
35	E	1.429	1.444	0.63	2.014	0.643
36	A	1.108	1.313	1.021	2.843	0.694
37	D	1.582	1.337	0.486	2.067	0.662
38	B	1.238	1.439	0.881	2.357	0.721
39	B	0.744	1.053	1.585	4.909	0.701
40	B	1.145	1.366	0.965	2.613	0.719
41	A	1.11	1.42	1.015	2.598	0.615
42	B	1.307	1.375	0.794	2.363	0.725
43	D	0.82	1.085	1.494	4.627	0.534
44	B	1.183	1.301	0.892	2.599	0.647
45	D	1.324	1.338	0.727	2.322	0.713
46	B	1.352	1.376	0.759	2.314	0.715
47	B	1.441	1.359	0.62	2.136	0.642
48	A	1.201	1.316	0.861	2.537	0.714
49	B	0.579	1.062	1.973	6.066	0.669
50	C	1.737	1.413	0.335	1.819	0.813
51	B	0.749	1.227	1.554	4.171	0.704
52	B	0.475	0.928	2.267	7.809	0.646
53	E	1.363	1.307	0.703	2.364	0.408
54	B	0.985	1.278	1.191	3.253	0.78
55	D	0.957	1.164	1.234	3.673	0.551
56	B	0.576	1.065	2.008	6.204	0.67
57	E	1.121	1.236	0.973	2.918	0.776
58	C	1.357	1.413	0.713	2.175	0.53
59	D	0.996	1.167	1.168	3.539	0.478
60	D	1.016	1.241	1.101	3.136	0.562
61	E	1.738	1.396	0.307	1.83	0.513
62	B	0.994	1.233	1.227	3.461	0.623
63	A	1.301	1.307	0.783	2.473	0.858
64	A	1.317	1.328	0.741	2.362	0.888
65	A	1.174	1.375	0.915	2.528	0.558
66	C	1.711	1.563	0.353	1.588	0.704
67	E	1.734	1.365	0.341	1.889	0.877
68	E	1.735	1.47	0.307	1.704	0.876
69	E	1.762	1.425	0.274	1.747	0.48
70	E	2.23	1.46	−0.134	1.627	0.332

M, Mean; SD, Standard deviation; γ1, skewness; γ2, excess kurtosis; λ, standardized factor loading; ω, reliability coefficient; A, Stimulus-related anxiety and avoidance behavior; B, Social anxiety and cognition of mobbing; C, Health- and body-related anxieties; D, Cognition of insufficiency; E, Jobrelated worrying.

Thereafter, we tested the measurement invariance of the 5-factor model across participant sex and age. Our results showed clear evidence for strict invariance across age groups, but not for sex. The results of these analyses are reported in Table 3.

**Table 3:** Fit indices for the analysis of measurement invariance.

Model	*χ*^2^(*df*)	Δ*χ*^2^	Δ*df*	*p*	*CFI*	Δ*CFI*	*RMSEA*	Δ*RMSEA*
Sex								
Multigroup analysis								
Configural invariance	15368.273 (4670)				0.746		0.080	
Metric invariance	15467.917 (4735)	99.644	95	0.352	0.746	0.000	0.079	0.001
Scalar invariance	15690.372 (4800)	222.455	65	<0.001	0.744	0.002	0.079	0.000
Strict invariance	15758.134 (4870)	67.762	70	0.553	0.744	0.000	0.079	0.000
Age, years								
Multigroup analysis								
Configural invariance	17920.793 (7005)				0.746		0.081	
Metric invariance	18121.398 (7135)	200.605	130	<0.001	0.746	0.000	0.080	0.001
Scalar invariance	18914.762 (7265)	793.364	130	<0.001	0.736	0.010	0.081	0.001
Strict invariance	19262.775 (7405)	347.993	140	<0.001	0.731	0.005	0.081	0.000

*χ*^2^, scaled chi square statistic; CFI, robust comparative fit index; GH, scaled gamma hat. For participant sex, the analysis was only conducted in the confirmatory sample to avoid statistical dependence with the exploratory analysis.

Next, we examined the convergent validity of the JAS (see Table 4). In this regard, we found the expected pattern of correlations with the HEALTH-49. Namely, symptoms of mental distress and social restrictions correlated positively high with job anxiety, and indicators of well-being and social integration evinced negative associations to the construct. To synthesize these results into a more comprehensive format we also ran a canonical correlation analysis. This yielded canonical correlation coefficients *R* of 0.620, 0.331, 0.231, 0.176, and 0.127 – with the first three being significant contributors (*p* < 0.001).

**Table 4:** Correlations between the JAS and the HEALTH-49.

	Somatoform	Depression	Phobia/Anxiety	Psychological and somatoform	Psychological well-being	Difficulties in interactions	Self-efficacy	Activity and participation	Social support	Social distress
A	0.287^**^	0.471^**^	0.456^**^	0.507^**^	−0.257^**^	0.402^**^	−0.393^**^	−0.283^**^	−0.150^**^	0.254^**^
B	0.246^**^	0.433^**^	0.389^**^	0.450^**^	−0.263^**^	0.437^**^	−0.333^**^	−0.252^**^	−0.148^**^	0.257^**^
C	0.358^**^	0.428^**^	0.410^**^	0.501^**^	−0.257^**^	0.334^**^	−0.376^**^	−0.298^**^	−0.115^**^	0.190^**^
D	0.327^**^	0.476^**^	0.423^**^	0.530^**^	−0.290^**^	0.420^**^	−0.424^**^	−0.297^**^	−0.119^**^	0.275^**^
E	0.347^**^	0.516^**^	0.398^**^	0.512^**^	−0.331^**^	0.433^**^	−0.396^**^	−0.298^**^	−0.142^**^	0.271^**^

JAS, Job Anxiety Scale; HEALTH-49, Hamburg Modules for the Assessment of Psychosocial Health; A, Stimulus-related anxiety and avoidance behavior; B, Social anxiety and cognition of mobbing; C, Health-and body-related anxieties; D, Cognition of insufficiency; E, Job-related worrying; ^**^Significant at *p* < 0.01.

## Discussion

The aim of the present study was to reevaluate the psychometric properties of the JAS and additionally confirm its factor structure. In contrast to other scales (e.g., WPS), the JAS is an empirically-derived extract which assess job related anxiety symptoms in a comprehensive manner.

In the present study, the JAS was found to be highly reliable. This result is in line with the research conducted by Linden et al. (2008) who found very good values for Cronbach’s alpha (α = 0.98) as well. Apart from the JAS being a reliable scale, we also found evidence for its strict measurement invariance across age groups, but not for sex. This means that group means across ages can be meaningfully compared and inferences can be drawn from these comparisons, which is a useful property in this scale. From the latter result it can be drawn, that men and women rate their job anxiety differently and their scores should be evaluated with this in mind. Despite these positive results, the confirmatory analysis, revealed a 5-factor structure with an unacceptable fit, as evidenced by low *CFI* and *TLI* values. This means that even if the measurement process is comparable between age and gender-groups, basic factorial validity is not given. This results from correlated measurement errors and could be rectified in a revised scale.

A strength of the present study is the very large sample of participants and the measurement invariance of the scale in terms of age, which enhances the quality of our results. Moreover, the JAS and its subscales displayed convergent validity with a measure of psychosocial health in the expected manner. The JAS correlated positively with depression, phobia/anxiety and psychological somatoform symptoms and negatively with social support and well-being, self-efficacy and activity/participation (see Table 4), which coincides with previous findings (Hobson and Beach, 2000; Greenglass, 2002; Muschalla et al., 2013; Huhtala et al., 2021).

It should be noted that the JAS correlated roughly equally (*r* ~ 0.30–0.40) with the phobia/anxiety subscale of the instrument and with other measures of psychological distress. This apparent “lack” of differential correlation patterns between the different forms of psychological distress can be explained by the fact that the phobia/anxiety subscale of the HEALTH-49 is focused on classic phobia symptoms such as agoraphobia and specific phobias (e.g., fear of elevators).

A useful application of the scale concerns the field of occupational psychological and practice. As an example, the JAS can be used for risk assessment in the context of incapacity for work, high termination rates, risk assessment of unemployment in the context of medical rehabilitation. Further the JAS can be used to assess conflicts in the work-place and improve working environment, reduce rates of terminations and improve performance at work. Future studies should differentiate these aspects concerning sex and gender and reveal differences between men and women and how they cope with job anxiety related concerns in order to shed light in this field. Moreover, it is recommended to adjust further scales concerning invariance in this matter.

### Limitations

Since the JAS is a self-report scale the validity of the assessment is tied to the individuals responding to it. Among other works, Razavi (Razavi, 2001) discusses the shortcomings of these measures – such as acquiescence and social desirability – as well as potential remedies.

The study is based on data collected in a clinical environment with a large proportion of psychosomatic and rehabilitation patients. Therefore, it appears questionable that the results can be transferred without reservation to other clinical and nonclinical populations. Additionally, the sample consisted of 991 patients from two different clinics. A larger sample size from more clinics – or even from the general population – would provide an even wider database. Only little research has been carried out concerning job-related anxiety. Usually, researchers adapt different instruments or constructs in order to measure job anxiety. Therefore, further research in clinical and nonclinical samples will be necessary in order to understand the underlying construct of job-related anxiety. Based on this knowledge, the JAS should be subject to further testing and be further developed. Also, so far it is still unclear how sensitive the JAS might react to changes in a person or an organization. Therefore, the sensitivity of the JAS regarding changes should be tested. Due to its length and its unacceptable fit, future researches could focus on shortening the scale for a more economic assessment and perhaps a better fit.

## Conclusion

The aim of this study was to evaluate the psychometric properties of the empirically derived JAS and run a confirmatory analysis, which has been missing in past studies. Our analysis revealed satisfactory psychometric properties but did not confirm the 5-factorial structure. Thus, future studies could focus on shortening the scale and aim a better fit in order to screen job related anxiety in a more efficient and valid manner (REV4).

## Data availability statement

The raw data supporting the conclusions of this article will be made available by the authors, only by reasonable request.

## Ethics statement

The studies involving human participants were reviewed and approved by Technische Universität, Dresden (EK 79032011). The patients/participants provided their written informed consent to participate in this study.

## Author contributions

KP and MB provided data and supervised the process of creating this paper. AK contributed substantially to conception and design. All authors have made substantial contributions to analysis and interpretation of data. IS and BS executed the statistical analyses. IS and BS wrote the manuscript. All authors revised it critically for important intellectual content, and read and approved the final manuscript.

## Conflict of interest

The authors declare that the research was conducted in the absence of any commercial or financial relationships that could be construed as a potential conflict of interest.

## Publisher’s note

All claims expressed in this article are solely those of the authors and do not necessarily represent those of their affiliated organizations, or those of the publisher, the editors and the reviewers. Any product that may be evaluated in this article, or claim that may be made by its manufacturer, is not guaranteed or endorsed by the publisher.

## Abbreviations

JAS: Job Anxiety Scale
WPS: Workplace Phobia Scale
CFI: Comparative Fit Index
TLI: Tucker-Lewis Index
RMSEA: Root Mean Square Error of Approximation
SRMR: Standardized Root Mean Square Residual
ICD: International Statistical Classification of Diseases and Related Health Problems
DSM: Diagnostic and Statistical Manual of Mental Disorders
STAI-T: State-Trait-Anxiety Inventory
HEALTH: Hamburg Modules for the Assessment of Psychosocial Health
